# Extirpation of Gland

**Published:** 1863-06

**Authors:** 


EXTIRPATION GLAND.—*Wheeling, West Virginia, May 30th,* 1863.—*Editor Medical and Surgical Reporter*:—On the 10th of January last, Mrs. C., aged 45 years, native of Ireland, of good general health, though of cachectic appearance, residing in this city, presented herself at my office for advice in relation to a tumor below the right ear, which first made its appearance some ten years since. When first discovered, it was not larger than a pea, but gradually increased until three years since. She consulted Dr. Housten, then of this city, who (she says) passed a seaton through the tumor for the purpose of reducing it by suppuration; he failed however. Since that time it has been steadily increasing in size, accompanied with occasional lancinating pains. At present it occupies all the space between the mastoid process and the angle of the jaw, thrusting the ear upward, is irregular or knotty on the surface, having clefts and globular projections, and is almost immoveable; protruding but little externally, but seems to dip down deeply, compressing the parts beneath, and is of almost stony hardness, seeming to implicate the greater part of the parotid gland.

My diagnosis was scirrhus of the parotid gland, and I proposed its removal by an operation. After having fully stated the difficulty and risk attending such an operation to my patient and her husband, she decided at once upon its removal. Accordingly she was placed upon the operating chair, when I directed my assistant to render the surface tense, and proceeded to make a longitudinal incision extending from the mastoid process to the ascending portion of the inferior maxillary. I then made a crucial incision and dissected back the flaps, so as to expose the entire surface of the tumor. I then opened the fibrous capsule and cautiously proceeded to dissect out the tumor after the method of Professor Chelius, securing the arteries as I progressed, sometimes using the knife, sometimes the handle of the scalpel and sometimes my fingers alone, my assistant lifting the tumor from its bed with a tenaculum, which was in such close proxmity to the carotid artery as to present after its removal a groove or depression through which that vessel passed. I brought the wound together, secured the flaps by stitches, and applied cold water dressing. The patient bore this tedious and painful operation with the utmost fortitude, scarcely uttering a murmur. Considerable blood continued to ooze from the wound during the early part of the night which was staunched by the application of ice. After the administration of an opiate, the patient, although somewhat nervous, obtained some sleep.

The case continued to progress favorably, the ligatures came away, and in three weeks from the operation, cicatrization is complete.

To-day, May 30th, 1863, no trace of the disease is perceptible, the scar produces but little deformity and the patient only complains of a very slight numbness or want of feeling in the neighboring parts produced by interruption of nervous communication. Her general health is much improved. E. Halley McCoy.

MUSCATINE, IOWA, June 10, 1863.

DEAR SIR:—We, the undersigned, Dentists of Iowa, feeling that in order to keep pace with the Dental profession in other States, and especially in order to lend our aid in its advancement as a profession, must labor unitedly, and for the purpose of putting ourselves in a position where we can do so, we very earnestly invite all Dentists in Iowa to meet with us in Convention at Muscatine, Iowa, on Tuesday evening, July 14, at 7 o'clock P.M., and there organize a State Dental Society, elect delegates to the American Dental Association, agree upon a list of prices, talk over matters pertaining to the best means of advancing our noble profession, &c., &c.

We are invited to bring reports of difficult cases in daily practice, and thus let us try to benefit each other. Let every Dentist in Iowa come and spend a few days in thus conferring with each other, and we can assure him he will be doubly paid for his time.

Arrangements have been made with the Hotels of the city to board members of the Convention at reduced rates.

Hoping you will not fail to come, we are

Respectfully yours,

DR. COULSON....Iowa City

" TULLOSS.... "

" MEYERS....Davenport

" NEWELL.... "

" HARRIS.....Rock Island, Ill.

" KULP ......Muscatine.

" HARDMAN... "

DRS. BROWNSON &

McCOLLOM..Burlington.

DR. HICKS.......Keokuk.

" TOOF .......Ft Madison.

DR. KEARNE...Mt Pleasant.

" MOREHEAD, "

" OGDEN ....Washington

" DEWEY.... "

" KENNEDY..Clay.

" RAWSON ...Des Moines.

" BROWNELL. "

" CHASE....Independence

" CLARKE.... Dubuque.

" McCAULEY, CedarRapids

" HUNT......McGregor.

